# Diagnostic accuracy of DNA methylation for head and neck cancer varies by sample type and number of markers tested

**DOI:** 10.18632/oncotarget.12219

**Published:** 2016-09-23

**Authors:** Xu Ji, Chao Guan, Xuejun Jiang, Hong Li

**Affiliations:** ^1^ Department of Otolaryngology, The First Affiliated Hospital of China Medical University, Shenyang, 110001, China; ^2^ Department of Otorhinolaryngology Head and Neck Surgery, The Fourth Affiliated Hospital of China Medical University, Shenyang, 110032, China

**Keywords:** head and neck cancer, DNA methylation, diagnostic accuracy, biopsy type, meta-analysis

## Abstract

Abnormal methylation of certain cancer related genes strongly predicts a diagnosis of head and neck cancer (HNC), while the predictive power of methylation of other DNA markers for HNC remains unclear. To systemically assess the diagnostic value of DNA methylation patterns for HNC and the effect of methylation platform techniques and sample types, we performed a PubMed search for studies of the correlation between DNA methylation and HNC completed before July 2016, and extracted the sensitivity and specificity for methylated biomarkers. Across these studies, DNA methylation showed high sensitivity for diagnosing HNC in solid tissue (0.57), and high specificity in saliva (0.89). Area under the curve (AUC) from summary receiver operating characteristic (SROC) curves revealed that DNA methylation had more diagnostic power in solid tissue (AUC = 0.82) than saliva (AUC = 0.80) or blood (AUC = 0.77). Combinations of multiple methylated genes were more sensitive diagnostic markers than single methylated genes. Our results suggest that the diagnostic accuracy of methylated biomarkers for HNC varied by sample type and were most accurate when results from multiple sample types were considered.

## INTRODUCTION

For this study, we selected from the literature reports of common squamous-cell carcinomas of the oral cavity, pharynx, and larynx (HSCC), which account for 90% of HNC [1]. Approximately 650,000 cases of HNC are identified per year [2] and the disease has high recurrence rates and poor prognoses due to distant metastasis [3]. Late diagnosis results in poorer prognosis [4]. Improved diagnostic accuracy for HNC could lead to earlier diagnosis, increasing patient survival rates.

Variations in the epigenetic modifications, such as DNA methylation in gene promoters, often inhibit gene transcription and protein translation, important factors in human carcinogenesis. A number of genes are frequently methylated in HNC, including *p16*, *DAPK1*, and *RASSF1A* [5, 6], or hypermethylated in CpG islands, such as *hMLH1* [7], *KIF1A*, and *EDNRB* [8]. Many groups have identified abnormally methylated genes as HNC diagnostic biomarkers but their predictive accuracies fluctuated among different sample types. Moreover, there are no systematic diagnostic accuracy studies or meta-analyses regarding the various sample types in HNC. We performed a systematic review and stratified meta-analysis of previous HNC studies based on sample types and diagnostic markers. We aim to provide more reliable evidence to clarify the diagnostic accuracy of DNA methylation markers, according to published reports that computed sensitivity and specificity.

## RESULTS

### Study characteristics

**Figure 1:**
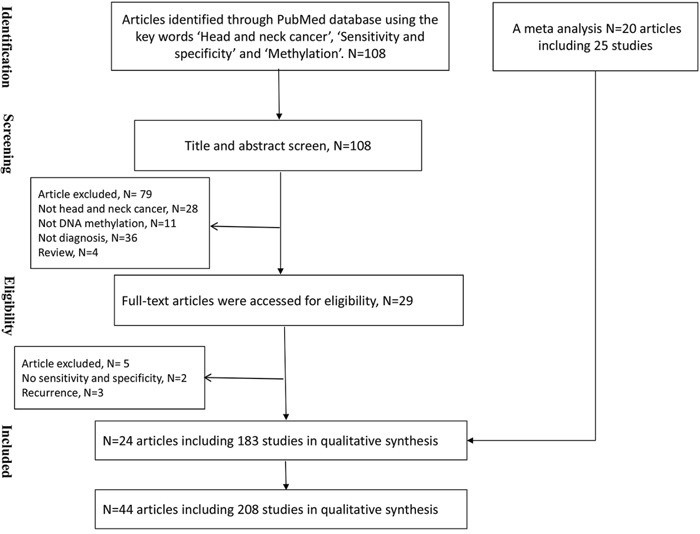
Flow chart showing the study retrieval process

We identified 108 papers in a search of the PubMed database. Seventy-nine were excluded based on screening the title and abstract, including twenty-eight papers that did not involve HNC, thirty-six papers that did not investigate the cancer diagnoses, eleven papers that did not include a diagnosis based on DNA methylation, and four reviews. We obtained the full texts of twenty-nine papers; of these five further papers were excluded, including two studies that did not show the sensitivity and specificity of the methylation biomarkers in a HNC diagnosis and three studies that only investigated the diagnosis of recurrence. We identified 183 studies from the remaining twenty-four articles [8–31] (Figure 1). In addition, we added 20 articles including 25 studies to our analysis from a review [32–51]. These studies were conducted in fifteen countries or regions (including the USA, Brazil, China, Hong Kong, Japan, Australia, Sweden, Egypt, Thailand, India, Taiwan, Hungary, Turkish, French and Italy) and were published between 2002 and 2016. The sample sizes of these studies ranged from 31–597 patients, with a mean of 115.

The diagnostic accuracy of selected methylated genes was extracted from the included papers and grouped by sample type tested. Ten papers used saliva [9, 10, 12, 14, 17, 27, 29, 32, 50], sixteen papers used solid tissue [11, 15, 16, 18, 19, 23–26, 30, 31, 33–38, 40–46, 49], four papers used blood [21, 28, 31, 39], five papers used both solid tissue and saliva [8, 13, 22, 47, 51], and two papers used both solid tissue and blood (Table 1) [20, 33]. The studies evaluated the diagnostic power of methylation biomarkers as follows: thirty-five studies were based on a single gene [8, 12–14, 16–19, 21, 23, 25–28, 30, 32–51], two papers were based on multiple genes [9, 22] and seven papers were based on both single and multiple genes [10, 11, 15, 20, 24, 29, 31]. The details of methylated biomarkers and their diagnostic powers are shown in Supplementary Table 1.

**Table 1:** The included studies investigating the diagnosis of DNA methylation biomarkers in head and neck cancer

Study	Country	Case#	Control#	Sample	Biomarker	Technique	Methylated genes
Liu et al, 2016	China	246	246	Tissue	S	BeadChip	S100A8
Nawaz et al, 2015	Sweden	44	18	Tissue	S, M	MSP	EBNA1, LMP1, RASSF1A, DAPK, ITGA9, P16, WNT7A, CHFR, CYB5R2, WIF1, RIZ1, FSTL1
Arantes et al, 2015	Brazil	40	40	Saliva	S, M	qMSP	TIMP3, DCC, DAPK, CCNA1, AIM1, MGMT, CDH1, HIC1
Kis et al, 2014	Hungary	60	68	Saliva	S	MSP	P16
Bhatia et al, 2014	India	76	70	Tissue, Blood	S	MSP	P16
Dang et al, 2013	China	12	30	Tissue	S	MSP	P16
Puttipanyalears 2013	Thailand	88	161	Saliva	S	COBRA	ALU
Tian et al, 2013	China	40	41	Blood	S	MSP	RASSF1A, CDKN2A, DLEC1, DAPK1, UCHL1
Rettoriet al, 2013	Brazil	68	60	Tissue	S	BS	CCNA1, DAPK, MGMT, SFRP1, TIMP3
You et al, 2013	China	40	40	Blood	S	MSP, BS	CDK10
Schussel et al, 2013	USA	48	113	Saliva	S, M	qMSP	DCC, EDNRB
Ovchinnikov et al, 2012	Australia	143	31	Saliva	M	MSP	RASSF1A, p16, DAPK1
Minor et al, 2012	USA	59	48	Tissue	S, M	MSP	miR-9-1, miR-9-3
Nagata et al, 2012	Japan	34	24	Saliva	S	MSP	ECAD, TMEFF2, RARβ, MGMT, FHIT, WIF-1, DAPK, p16, HIN-1, TIMP3, p15, APC, SPARC
Zhange et al, 2012	Sweden	49	20	Tissue	S	MSP	EBNA1, LMP1, RASSF1A, DAPK
Demokan et al, 2011	Turkish	60	77	Tissue	S	MSP	P16
Li et al, 2011	China	47	15	Tissue	S, M	MSP	P16, DAPK, RARb, CDH1, RASSF1A
Weiss et al, 2011	Germany	51	31	Tissue	S	MSP	P16
Gyobu et al, 2011	Japan	40	8	Tissue	S	qMSP	PAX6, ENST00000363328
Loyo et al, 2011	Hong Kong	50	28	Tissue	S, M	qMSP	AIM1, APC, CALCA, DCC, DLEC, DLC1, ESR, FHIT, KIF1A, PGP9.5, TIG1
Guerrero-Preston et al, 2011	USA	24	12	Tissue, Saliva	S	BeadChip, qMSP	HOXA9, NID2, GATA4, KIF1A, EDNRB, DCC, MCAM, CALCA
Laytragoon et al, 2010	Sweden	41	18	Tissue	S	MSP	P16
Pattani et al, 2010	USA	48	113	Saliva	S	qMSP	EDNRB
Kaur et al, 2010	India	92	48	Tissue, blood	S	qMSP	P16
Tawfik et al, 2010	Egypt	34	15	Tissue	S	MSP	hMLH1
Su et al, 2010	Taiwan	30	30	Tissue	S	qMSP	P16
Cao et al, 2009	China	22	56	Tissue	S	MSP	P16
Steinmann et al, 2009	Germany	54	23	Tissue	S	MSP	P16
Ghosh et al, 2009	India	63	40	Tissue	S	MSRA	India
Viet et al, 2008	USA	13	23	Tissue, saliva	M	BeadChip	GABRB3, IL11, INSR, NOTCH3, NTRK3, PXN, ERBB4, PTCH2, TMEFF1, TNFSF10, TWIST1, ADCYAP1, CEBPA, EPHA5, FGF3, HLF, AGTR1, BMP3, FGF8, NTRK3, FLT, IRAK3, KDR, NTRK, RASGRF1, WT1, ESR1, ETV1, GAS7, PKD2, WNT2, EPHA5, GALR1, KDR, p16, AGTR1, EYA4, IHH, NTRK3, NTRK3, TFPI2
Adams et al, 2008	USA	51	50	Tissue, blood	S, M	qMSP	AHRR, p16, CBRP, CLDN3, MT1G, MGMT, RARβ, PGP9.5
Carvalho et al, 2008	USA	135	462	Tissue, saliva	S	qMSP	DCC, DAPK, ESR, CCNA1, CCND2, MINT1, MINT31, CDH1, AIM1, MGMT, p16, PGP9.5, RARβ, HIC1, RASSF1A, CALCA, TGFBR2, S100A2, RIZ1, RBM6
Righimi et al, 2007	French	90	30	Tissue, saliva	S	MSP	P16
Franzmann et al, 2007	USA	102	69	Saliva	S	MSP	CD44
Martone et al, 2007	Italy	20	11	Tissue	S	MSP	P16
Shaw et al, 2006	UK	80	26	Tissue	S	Pyro	P16
Maruya et al, 2004	USA	14	32	Tissue	S	MSP	P16
Kulkarni et al, 2004	India	60	60	Tissue, saliva	S	MSP	P16
Weber et al, 2003	Germany	50	42	Tissue	S	MSP	P16
Wong et al, 2003	China	73	29	Tissue, blood	S	MSP	P16
Tong et al, 2002	Hong Kong	28	26	Tissue	S	MSP	EBV
Nakahara et al, 2001	Japan	32	32	Tissue	S	MSP	P16
Rosas et al, 2001	USA	30	30	Saliva	S	MSP	P16
Sanchez et al, 2000	USA	95	26	Blood	S	MSP	P16

S represented single methylated gene as diagnostic marker, and M represented combination of multiple methylated genes as diagnostic marker.

### Exploration of heterogeneity analysis

To determine the effect model of diagnostic accuracy, we conducted heterogeneity tests for PLR, NLR, and DOR and found a significant heterogeneity of NLR in the solid tissue and saliva studies (Table 2). DOR showed no heterogeneity in the solid tissue or blood studies. PLR showed low heterogeneity in the solid tissue studies and no heterogeneity in the saliva or blood studies. The heterogeneity of NLR varied among the sample types.

**Table 2:** Heterogeneity analysis of diagnostic effects

Sample	Effects	Estimate[95% CI]	Log(Estimate) [95% CI]	df	Q	P-value	I^2^
All	PLR	3.45[3.07-3.88]	1.24[1.12-1.35]	207	257.16	0.01	19.51%
	NLR	0.62[0.59-0.64]	−0.48[-0.52 to −0.44]	207	578.57	<0.01	64.22%
	DOR	7.84[6.56-9.35]	2.06[1.88-2.24]	207	242.98	0.044	14.81%
Saliva	PLR	3.60[2.97-4.37]	1.28[1.09-1.47]	75	63.44	0.827	0%
	NLR	0.71[0.67-0.74]	−0.35[-0.40 to −0.30]	75	290.26	<0.01	74.16%
	DOR	6.84[5.45-8.59]	1.92[1.70-2.75]	75	106.90	0.01	29.84%
Tissue	PLR	3.85[3.08-4.83]	1.35[1.13-1.57]	70	81.91	0.156	14.54%
	NLR	0.52[0.47-0.57]	−0.66[−0.76 to −0.56]	70	117.57	<0.01	40.46%
	DOR	10.96[7.57-15.89]	2.40[2.02-2.77]	70	68.33	0.534	0%
Blood	PLR	2.76[1.89-4.03]	1.01[0.63-1.39]	10	10.26	0.418	2.57%
	NLR	0.65[0.54-0.78]	−0.43[−0.61 to −0.25]	10	15.19	0.125	34.17%
	DOR	5.42[2.98-9.86]	1.69[1.09-2.29]	10	8.28	0.60	0%

PLR: positive likelihood ratio. NLR: negative likelihood ratio. DOR: diagnostics odd ratio. Estimate [95% CI]: the pooled effect measure with the corresponding 95% confidence interval. Log (Estimate) [95% CI]: logarithmic transformation of the pooled effect measure with the corresponding 95% confidence interval. df: degrees of freedom. Q and P-value were the Q value and p value of Cochran's Q test.

We used meta-regression analysis to assess whether publication year, sample type, DNA methylation detection technique, or the methylation panel corresponding to single or multiple methylated biomarkers affected the diagnostic accuracy for HNC. The true and false positive rates were used as the responses in meta-regression analyses. As shown in Table 3, the *p* values of sensitivity and false positive rates were not significant, suggesting that publication year, biomarker technique, and sample types did not affect the false positive rate.

**Table 3:** Meta-regression analysis

Factor	Sensitivity		False positive rate	
	Coefficient	p value	Coefficient	p value
Year	0.132	0.070	0.018	0.559
Marker type	0.15	0.489	−0.193	0.379
Technique	−0.185	0.051	−0.023	0.824
Sample	0.068	0.345	0.098	0.186

### Meta-analysis and diagnostic accuracy

**Figure 2:**
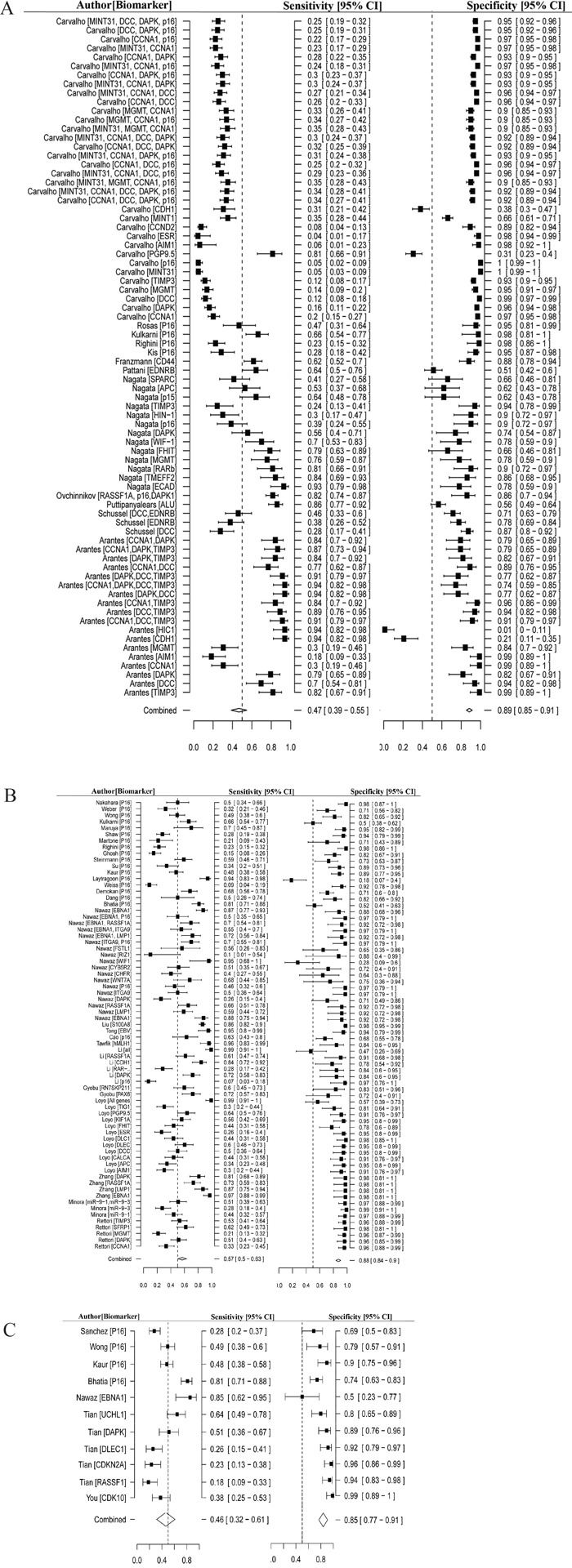
Forest plot of estimate of diagnostic accuracy using methylated biomarkers **A.** Forest plot of estimate of sensitivity and specificity of methylated biomarkers in saliva. **B.** Forest plot of estimate of sensitivity and specificity of methylated biomarkers in tissue. **C.** Forest plot of estimate of sensitivity and specificity of methylated biomarkers in blood.

The pooled sensitivity and specificity of meta-analysis was 0.52 (95% CI 0.47-0.57) and 0.87 (95% CI: 0.85-0.89), respectively. Meta-analysis was performed separately for the saliva, solid tissue, and blood samples. DNA methylation detected from saliva samples had an overall sensitivity and specificity for HNC diagnosis of 0.47 (95% CI: 0.39-0.55) and 0.89 (95% CI: 0.85-0.91), respectively (Figure 2A). In solid tissue samples the overall sensitivity and specificity were 0.57 (95% CI: 0.50-0.63) and 0.88 (95% CI: 0.84-0.9), respectively (Figure 2B). Blood samples provided the lowest overall sensitivity at 0.46 (95% CI: 0.32-0.61), and overall specificity of 0.85 (95% CI: 0.77-0.91, Figure 2C).

In addition, we evaluated the diagnostic power based on the types of methylation biomarkers. The single methylation markers had overall sensitivity and specificity of 0.51 (95% CI: 0.45-0.57) and 0.87 (95% CI: 0.84-0.90), respectively. The diagnostic sensitivity of multiple methylation markers was 0.55 (95% CI: 0.47-0.63) and the specificity was 0.88 (95% CI: 0.85-0.90). In general, methylated biomarkers showed differential diagnostic accuracy in all three sample types, and the diagnostic power of integrating multiple methylated genes was better than with a single gene.

**Figure 3:**
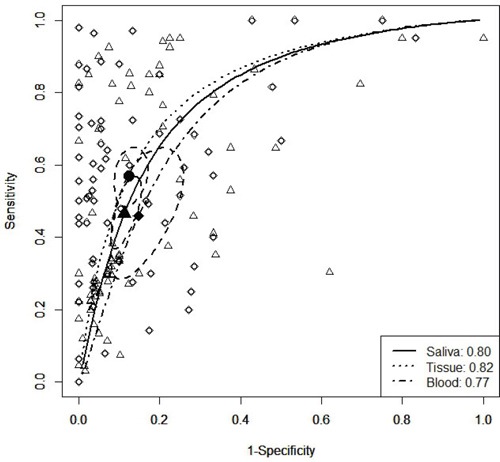
SROC curves of studies relating to the detection of HNC in different biopsy types

According to the sensitivity and specificity results from each trial, the regression coefficients of the SROC curves were near 0 for the three sample types (Table 4). The AUC curve indicated that samples from solid tissue had the highest diagnostic accuracy, with an AUC value of 0.82 (Figure 3) and a Q* metric of 0.81 (95% CI: 0.77–0.85), whereas the sensitivity was identical to the specificity (Table 4, Figure 3). In addition, the panel of multiple methylated genes showed higher AUC value than a single methylated gene (0.85 vs. 0.77). These results suggest that the combination of multiple methylation biomarkers in solid tissue has better diagnostic accuracy, with higher sensitivity in saliva, which could be useful for HNC screening.

**Table 4:** The main analysis results of SROC

Sample	Sensitivity (95% CI)	Specificity (95% CI)	a (95% CI)	b (95% CI)	AUC	Q* (95% CI)
**Saliva**	0.47 (0.39 - 0.55)	0.89 (0.85 - 0.91)	2.14 (1.71-2.56)	−0.02 (-0.14-0.09)	0.80	0.74 (0.70-0.78)
**Tissue**	0.57(0.5 - 0.63)	0.88(0.84 - 0.90)	2.91 (2.37 - 3.44)	0.07 (−0.11 - 0.25)	0.82	0.81 (0.77-0.85)
**Blood**	0.46 (0.32 - 0.61)	0.85 (0.77 -.91)	1.75 (0.,74 - 2.77)	−0.09 (−0.42 - 0.24)	0.77	0.71 (0.59 - 0.80)

Sensitivity and Specificity represent the independent pooled sensitivity (Se) and specificity (Sp) using fixed effect model. a and b represent the intercept and slope of SROC curve. AUC represent the area under SROC curve. Q* represents the diagnostic threshold at which the probability of a correct diagnosis is constant for all subjects and calculated as exp(a/2)/[1+exp(a/2)].

### Publish bias and sensitivity analysis

**Figure 4:**
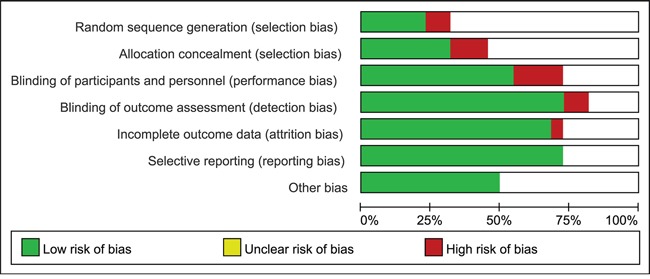
Risk of bias graph Two authors independently evaluated the items of bias. If the study reported all of the sensitivities and specificities of genes that were measured DNA methylation status, selective reporting was defined as low risk.

The risk of bias for each study was first assessed (Supplementary Figure 1). As shown in Figure 4, 92% of studies showed a low or unclear risk of bias for many bias items and only 5 ~ 18% of studies clearly reported a non-random sequence generation, no blinding, or incomplete blinding. More than 50% of the studies had independent data collection, assessment of DNA methylation, and interpretation of outcomes. In total, 75% of the studies show the sensitivities and specificities for all of the evaluated methylation biomarkers, which suggested no selective reporting. Ten studies were reported to be free of other sources of bias. Based on these metrics we deemed the quality of the studies included in the following meta-analysis to be acceptable.

**Figure 5:**
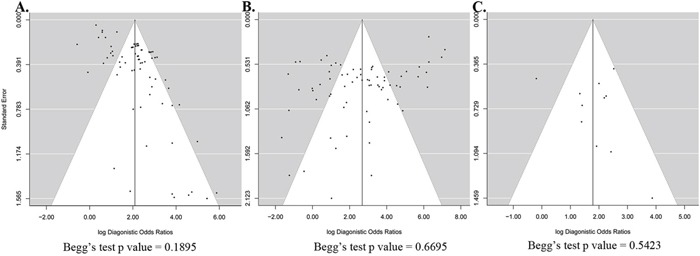
Funnel plot to assess bias in estimates of diagnostic odds ratio caused by small-study effects **A.** Saliva. **B.** Solid tissue. **C.** Blood.

By testing the relationship between the DOR and its standard error, we assessed the publication bias effects of the sample size for each diagnostic consequence. The potential publication bias was ascertained in these studies using symmetrical funnel plots for the saliva, solid tissue, and blood samples. We found that some studies corresponding to saliva (Figure 5A) or solid tissue (Figure 5B) were not inside the funnel. Begg's testing demonstrated that there was no significant publication bias in the three sample types from HNC patients. The studies with smaller sample sizes did not tend towards higher levels of accuracy.

A one-way sensitivity analysis was performed to evaluate the robustness of the results of this meta-analysis with respect to study and biomarker. The pooled specificity was not influenced when removing one study or diagnostic biomarker (Supplementary Table 2, 3). The sensitivity was increased when the studies by Carvalho et al. and Adams *et al.* were excluded, and was decreased when the study by Arantes *et al.* was excluded (Supplementary Table 2). The exclusion of individual methylated markers had no effect on diagnostic sensitivity.

## DISCUSSION

DNA methylation has previously been demonstrated to be a potentially useful marker for multiple cancers [52]. Abnormally methylated regions in cancer-related genes such as *RASSF1A* [24], *p16*[53], *RAR-β*[24], and *MGMT* [54], provide adequate sensitivity and specificity for the detection of HNC. Other abnormally methylated genes have shown inconsistent diagnostic accuracy for HNC. For example, the sensitivity of *p16* for diagnosing HNC varied from 44.6% to 100% [19, 55].

In this study, we analyzed the accuracy of methylated genes for diagnosing HNC based on previously published studies. Overall, the sensitivity of the DNA methylation was 0.47 in saliva, 0.57 in solid tissue and 0.46 in blood, and the specificity was 0.89, 0.88 and 0.85, respectively. We found that DNA methylation had low sensitivity but high specificity in the diagnosis of HNC. Different samples showed similar specificity but differential sensitivity. Seven papers corresponding to eleven studies were used to estimate the diagnostic accuracy of DNA methylation in blood. The small number of studies may provide misleading conclusions on the diagnostic power in blood that should be further evaluated. Moreover, testing for multiple methylated genes showed higher sensitivity than single methylated genes. Ideally, we should assess the overall diagnostic accuracy of the same combinations of methylated genes or single genes in three different sample types, but were limited by the number of studies available in the literature. We provide detailed information on the combinations of multiple methylated genes in Figure 2. Behind each author's name is the applicable DNA methylation marker information for the specific study. The evidence from this study suggests that DNA methylation biomarkers might be effective tools for detecting HNC. It should also be noted that the diagnostic accuracy of DNA methylation depends on the sample type and diagnostic markers studied.

Many of the studies in our analysis detected DNA methylation based on methylation-specific PCR (MSP), one of the principle methods of investigating DNA methylation. MSP typically overestimates the extent of methylation, which would affect the diagnosis of HNC. We studied whether the assay method of DNA methylation affects the HNC diagnosis, but found no significant differences among these methods.

## MATERIALS AND METHODS

### Data sources and search strategy

We searched for diagnostic studies in PubMed published before July 2016. The search strategy for PubMed was (“head and neck neoplasms” [MeSH Terms] OR “head and neck cancer” [All Fields]) AND (“sensitivity and specificity”[MeSH Terms] OR “sensitivity and specificity” [All Fields] AND (“DNA methylation” [MeSH Terms] OR “DNA methylation” [All Fields]) to find appropriate studies published in English prior to July 25, 2016. We searched for published trials that evaluated the diagnostic accuracy of one or more methylated biomarkers. In addition, we added all studies included in a previous meta-analysis into our analysis [56].

### Study selection

Two reviewers independently filtered the search results by the title and abstract. Studies were excluded if they did not pertain to DNA methylation, were not related to HNC, were not diagnostic studies, or were reviews. Two authors obtained the full text of each paper and further filtered out the studies that did not supply sensitivity or specificity data for HNC diagnosis or that concerned the diagnosis of recurrence. We collected the authors' names; institutions; publication dates; sample types, including saliva, solid tissue, and blood; methylated biomarkers; and techniques of DNA methylation detection for all of the studies. All studies were evaluated independently and discussed by the authors until any inconsistencies were resolved.

### Data extraction and quality assessment

A standardized data extraction form was used to extract the information from each paper including the first author, year of publication, country in which the study was performed, number of cases and controls, sample types, methylated gene names, DNA methylation detection techniques, number of methylated genes used as diagnostic biomarkers, and records of true positive, false positive, true negative and false negative results in head and neck cancer. Simultaneously, we evaluated the risk of bias according to pre-specified criteria from the Cochrane Collaboration's tool for assessing the risk of bias [57]. The two authors independently checked the risk of bias assessment for each trial using standardized methods including the following (Supplementary Table 4): whether a study showed selection bias including sequencing generation and allocation concealment; whether the performance was biased regarding the blinding of patients and study personnel; whether the detection was biased according to the assessment of the blinding of the outcome; and whether the attrition and reporting were biased by being based on incomplete outcome data and selective reporting, respectively.

### Data synthesis and statistical analysis

We extracted the number of true positive (TP), false positive (FP), true negative (TN) and false negative (FN) results based on the remaining studies. The summary effects of the positive likelihood ratio (PLR), negative likelihood ratio (NLR), diagnostic odds ratio (DOR), and 95% confidence intervals (CI) were further computed to estimate the statistical heterogeneities through Cochran's Q test, that approximately follows a χ^2^ distribution with k-1 degrees of freedom (where k is the number of included studies) [58]. We assessed I^2^ = ((Q-(k-1))/Q) ×100%, which ranged from 0–100%. I^2^ represents different degrees of heterogeneity, including low (0–25%), moderate (25–50%), high (50–75%) and very high (75–100%) [58]. The p value of the heterogeneity test determined whether a fixed- or random-effects model was used to estimate the diagnostic effects, and the significance level of heterogeneity was considered to be 0.05. The overall sensitivity and specificity were estimated to represent the diagnostic power of DNA methylation for the detection of head and neck cancer. For the overall diagnostic accuracy, an SROC curve was generated based on the sensitivity and specificity of each study using the equation D=a+b×S, where D = logit(Se) – logit(1-Sp) = log(OR) and S = logit(Se) +logit(1-Sp) [59]. In the regression equation, D represents the diagnostic power of the methylated biomarkers, and S represents the threshold of the classification between positive and negative. Because the parameters D and S are from different studies, the values of the regression coefficient closer to 0 suggested less significant heterogeneity in various studies, which corresponds to diagnostic accuracy. The area under the SROC curve (AUC) value was estimated to measure the overall diagnostic power of DNA methylation in individual studies. In addition, Q* = Se = 1-Sp was computed according to the regression equation of SROC, where Se = exp(a/2)/[1+exp(a/2)] and 1-Sp = 1/[1+exp(a/2)], which suggested that the diagnostic threshold for a correct diagnosis was constant for all of the subjects. Publication bias was evaluated using funnel plot analyses and Begg's and Egger's tests, with a significance level defined as 0.01. We used the *mada* and *metafor* package in R to performed the statistical analysis [60].

## SUPPLEMENTRAY MATERIALS










