# Supplementary material for: Diagnostic accuracy of DNA methylation for head and neck cancer varies by sample type and number of markers tested
Source: Oncotarget. 2016 Sep 23;7(48):80019–32. doi: 10.18632/oncotarget.12219 (PMC5346768; doi:10.18632/oncotarget.12219)
Supplement: Supplementary file 1 [file oncotarget-07-80019-s001.pdf]

Diagnostic accuracy of DNA methylation for head and neck cancer varies by sample type and number of markers tested

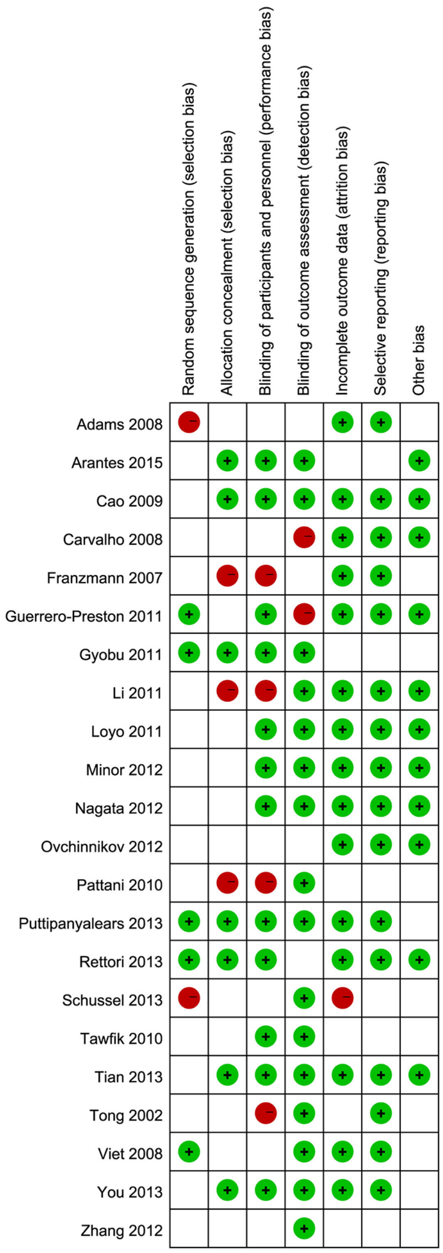

Supplementary Figure S1: Risk of bias summary.

Supplementary Table S1: Characteristics of all included studies and methylation biomarkers.

See Supplementary File 1

Supplementary Table S2: The one-way sensitivity analysis of studies.

See Supplementary File 2

Supplementary Table S3: The one-way sensitivity analysis of methylated marker.

See Supplementary File 3

Supplementary Table S4: The detailed of risk of bias table.

See Supplementary File 4
