# Supplementary material for: Diagnostic accuracy of DNA methylation for head and neck cancer varies by sample type and number of markers tested
Source: Oncotarget. 2016 Sep 23;7(48):80019–32. doi: 10.18632/oncotarget.12219 (PMC5346768; doi:10.18632/oncotarget.12219)
Supplement: Supplementary file 3 [file oncotarget-07-80019-s003.doc]

| Study | Sensitivity | Specificity | AUC |
| --- | --- | --- | --- |
| ALL | 0.52 | 0.87 | 0.79 |
| Adams | 0.54 | 0.87 | 0.80 |
| Arantes | 0.49 | 0.87 | 0.77 |
| Cao | 0.52 | 0.87 | 0.79 |
| Carvalho | 0.57 | 0.85 | 0.81 |
| Dang | 0.52 | 0.87 | 0.79 |
| Demokan | 0.52 | 0.87 | 0.79 |
| Franzmann | 0.52 | 0.87 | 0.79 |
| Ghosh | 0.52 | 0.87 | 0.79 |
| Gyobu | 0.52 | 0.87 | 0.79 |
| Kaur | 0.52 | 0.87 | 0.79 |
| Kis | 0.52 | 0.87 | 0.79 |
| Kulkart | 0.52 | 0.87 | 0.79 |
| Laytragoon | 0.52 | 0.87 | 0.79 |
| Li | 0.52 | 0.87 | 0.79 |
| Liu | 0.52 | 0.87 | 0.79 |
| Loyo | 0.52 | 0.87 | 0.79 |
| Martone | 0.52 | 0.87 | 0.79 |
| Maruya | 0.52 | 0.87 | 0.79 |
| Minora | 0.52 | 0.87 | 0.79 |
| Nagata | 0.51 | 0.88 | 0.79 |
| Nakahara | 0.52 | 0.87 | 0.79 |
| Nawaz | 0.51 | 0.87 | 0.79 |
| Ovchinnikov | 0.52 | 0.87 | 0.79 |
| Pattani | 0.52 | 0.87 | 0.79 |
| Preston | 0.52 | 0.87 | 0.79 |
| Puttipanyalears | 0.52 | 0.87 | 0.79 |
| Rettori | 0.52 | 0.87 | 0.79 |
| Righini | 0.52 | 0.87 | 0.79 |
| Rosas | 0.52 | 0.87 | 0.79 |
| Sanchez | 0.52 | 0.87 | 0.79 |
| Schussel | 0.52 | 0.87 | 0.80 |
| Shaw | 0.52 | 0.87 | 0.79 |
| Steinmann | 0.52 | 0.87 | 0.79 |
| Su | 0.52 | 0.87 | 0.79 |
| Tawfik | 0.52 | 0.87 | 0.79 |
| Tian | 0.52 | 0.87 | 0.79 |
| Tong | 0.52 | 0.87 | 0.79 |
| Viet | 0.52 | 0.88 | 0.79 |
| Weber | 0.52 | 0.87 | 0.79 |
| Weiss | 0.52 | 0.87 | 0.79 |
| Wong | 0.52 | 0.87 | 0.79 |
| You | 0.52 | 0.87 | 0.79 |
| Zhang | 0.52 | 0.87 | 0.79 |
