# Supplementary material for: Diagnostic accuracy of DNA methylation for head and neck cancer varies by sample type and number of markers tested
Source: Oncotarget. 2016 Sep 23;7(48):80019–32. doi: 10.18632/oncotarget.12219 (PMC5346768; doi:10.18632/oncotarget.12219)
Supplement: Supplementary file 5 [file oncotarget-07-80019-s005.doc]

Supplementary Table 4. Detailed of risk of bias table

| Bias | low risk | high risk | unclear risk | description |
| --- | --- | --- | --- | --- |
| random sequence generation | random generation of participants sequences | non-random component in the sequence generation process | Insufficient information | selection bias |
| allocation concealment | Participants and investigators could not foresee assignment | Participants and investigators could possibly foresee assignment | Insufficient information | selection bias |
| Blinding of participants and personnel | Blinding of participants or   outcome is not likely to be influenced by lack of blinding | No blinding or incomplete blinding   outcome is likely to be influenced by lack of blinding | Insufficient information | Performance bias |
| Blinding of outcome assessment | Blinding of outcome assessment or  outcome measurement is not likely to be influenced  by lack of blinding | No blinding of outcome assessment  the outcome measurement is likely to be influenced by lack of blinding | Insufficient information  not address this outcome | detection bias |
| Incomplete outcome data | No missing outcome data or  Reasons for missing outcome data unlikely to be related to true outcome | Reason for missing outcome data likely to be related to true outcome | Insufficient reporting | attrition bias |
| Selective reporting | The study protocol is available and all of outcomes that are of interest in the review have been reported in the pre-specified way; | Not all of the study’s pre-specified primary outcomes have been reported; | Insufficient information | reporting bias |

The items of bias were independently evaluated by two authors. If the study clearly reported the statement described in ‘low risk’ , the items was defined as low risk. If there was evidence showed the statement described in ‘high risk’ in the study, it was defined as high risk, otherwise we defined as unclear risk. If the study reported the sensitivity and specificity of all detected methylated genes, selective reporting was defined as low risk.
